# Mangrove forest classification and aboveground biomass estimation using an atom search algorithm and adaptive neuro-fuzzy inference system

**DOI:** 10.1371/journal.pone.0233110

**Published:** 2020-05-21

**Authors:** Minh Hai Pham, Thi Hoai Do, Van-Manh Pham, Quang-Thanh Bui

**Affiliations:** 1 Vietnam Institute of Geodesy and Cartography, Ha Noi, Viet Nam; 2 Center for Applied Research in Remote Sensing and GIS (CARGIS), Faculty of Geography, VNU University of Science, Vietnam National University, Hanoi, Thanh Xuan, Ha Noi, Viet Nam; Pablo de Olavide University, SPAIN

## Abstract

**Background:**

Advances in earth observation and machine learning techniques have created new options for forest monitoring, primarily because of the various possibilities that they provide for classifying forest cover and estimating aboveground biomass (AGB).

**Methods:**

This study aimed to introduce a novel model that incorporates the atom search algorithm (ASO) and adaptive neuro-fuzzy inference system (ANFIS) into mangrove forest classification and AGB estimation. The Ca Mau coastal area was selected as a case study since it has been considered the most preserved mangrove forest area in Vietnam and is being investigated for the impacts of land-use change on forest quality. The model was trained and validated with a set of Sentinel-1A imagery with VH and VV polarizations, and multispectral information from the SPOT image. In addition, feature selection was also carried out to choose the optimal combination of predictor variables. The model performance was benchmarked against conventional methods, such as support vector regression, multilayer perceptron, random subspace, and random forest, by using statistical indicators, namely, root mean square error (RMSE), mean absolute error (MAE), and coefficient of determination (R^2^).

**Results:**

The results showed that all three indicators of the proposed model were statistically better than those from the benchmarked methods. Specifically, the hybrid model ended up at RMSE = 70.882, MAE = 55.458, R^2^ = 0.577 for AGB estimation.

**Conclusion:**

From the experiments, such hybrid integration can be recommended for use as an alternative solution for biomass estimation. In a broader context, the fast growth of metaheuristic search algorithms has created new scientifically sound solutions for better analysis of forest cover.

## Introduction

Biomass, which includes above- and belowground biomass, is a critical component of carbon budget accounting and carbon monitoring, especially under the context of climate change [1,2]. Aboveground biomass (AGB) includes both live and dead material; estimation of the AGB of live trees has been more prominent in recent research. Accurate biomass quantifications are a prerequisite for a better understanding of the impacts of deforestation and environmental degradation on climate change [3]. The estimation of biomass is now crucial, as it is considered an essential source of energy in many countries. Technically, there are two biomass estimation methods. The destructive methods require tree cutting and further indoor weighing procedures [4]. These methods are limited to smaller areas and are usually employed to measure the biomass of sample plots that can also be used as ground truth samples. On the other hand, non-destructive measures take advantage of spatial technology to estimate AGB from a distance through backscatter or reflectance signals [1].

Mangrove forests cover a small portion of the global land area [5], but their carbon-rich ecosystems play a crucial role in sustaining the livelihoods of coastal communities [6], protecting coastal lines and inner land from storms and tsunamis [7], offsetting anthropogenically produced carbon dioxide, and contributing to carbon export to the ocean [8]. However, mangrove forests are threatened by human interactions that claim forest cover for aquaculture and agricultural activities. The estimated global loss of mangrove forests is approximately 0.16 to 0.39% [9], which poses a significant risk to the total carbon emission rate because of the considerable proportion of carbon storage in mangrove forests [9,10]. The surveillance of mangrove biomass is, therefore, crucial for estimating the potential carbon stored in these forests for global emission reduction programs.

Remote sensing (optical, radio detection and ranging- radar; light detection and ranging- Lidar) probably provides the best alternative for estimating AGB on a large scale and enables repetitive and rapid assessment of biomass over large areas relatively quickly and at a low cost, providing a more spatially comprehensive measure of forest biomass variation. Radar remote sensing enables surveying operations in all weather conditions [11–13], and it is usually used in combination with optical imageries to obtain complementary information about mangrove structure and biomass [1,14]. Among the radar bands, AGB can be effectively calculated by using the high-frequency L-band and P-band because of their penetration capability, as explained in the studies of [1,11,15] through the use of ALOS PALSAR data. From a global perspective, both active and passive remote sensing have become vital sources of data for mapping the spatial distribution of vegetation [16,17].

There is a growing body of research on the application of remotely sensed imageries, machine learning algorithms, and geospatial information technology in AGB estimation [18,19]. The underlying scientific background is to understand the correlation between backscatter and the reflectance of remotely sensed data and live biomass over a given area [20,21]. The methods for AGB estimation are diverse. For example, the application of inversion of the PROSAIL model estimates AGB by using leaf dry matter content and leaf area index [22]. The combination of vegetation indices with radar data has also been investigated in several works [1,23]. From reviewing the literature, several types of research were found that employed machine learning models to estimate the AGB. As examples, statistical and data-driven approaches have been proposed, such as multiple regression in the study of [13,24], geographically weighted regression [25], and support vector regression and random forest in [1,23,24].

Recently, metaheuristic algorithms have gained considerable popularity because they are capable of searching for the optimal parameters of classifiers in image classification and disaster susceptibility mapping by solving objective functions that are differently defined case to case. Three typical types consist of physically based, swarm intelligence, and evolutionary algorithms that mimic the behaviors or mechanisms of natural events to mathematically model artificial applications [26–28]. New models are being examined for their simplicity, flexibility, and ability to solve complex nonlinear problems. However, few of these models can be found for biomass estimation in such a way that the optimization algorithm supports the improvement of the performance of the classifiers that are generally trained by conventional methods. Currently, new networks and models are continuously being developed for various applications, many of which are available as open-source libraries [13]. To the best of our knowledge, the use of metaheuristic algorithms in biomass studies is still limited.

Although there is a vast number of studies on applications of machine learning algorithms in biomass estimation, there are no models that fit all problems. Moreover, the search for optimal machine learning models is crucial to contribute to global knowledge in the field of forest management. This study aimed to investigate a novel combination of atom search optimization algorithms and adaptive neuro-fuzzy inference systems in classifying mangrove forests and estimating AGB. Ca Mau Province, a coastal area in southern Vietnam, was selected as a case study because of its diverse ecosystems and its role in protecting the coastal zone. This hybrid model was validated by using common statistical indicators, namely, root mean square error (RMSE), mean absolute error (MAE), and coefficient of determination (R^2^) and was benchmarked by regression models that had been used for mangrove studies, such as multilayer perceptron (MLP), support vector regression (SVR), random subspace (RS) and random forest (RF). The geospatial database was processed by Quantum GIS (QGIS), the segmentation process, and SPOT-6, and its derived indices were analyzed by using PCI Geomatica 2018 Service Pack 2. The Sentinel-1A imagery was processed by Sentinel Application Platform (SNAP) from the European Space Agency, and the model was coded in MATLAB R2018b. SVR, RF and multilayer perceptron were implemented in Weka version 8.3.

## Study area and data

### Description of the study area

In Vietnam, mangrove forests are mainly distributed in the northeastern and northern delta and the central and southern delta, with the densest area in the U Minh National Park. As the importance of mangrove forests has become widely recognized, the area has been regrown and expanded through national and international projects. However, the transformation of mangrove forests into aquaculture and other economic activities continues in some areas, and it makes the coastal zone more susceptible to natural hazards such as salinity intrusion, drought, coastal erosion and flooding [2,3].

**Fig 1:**
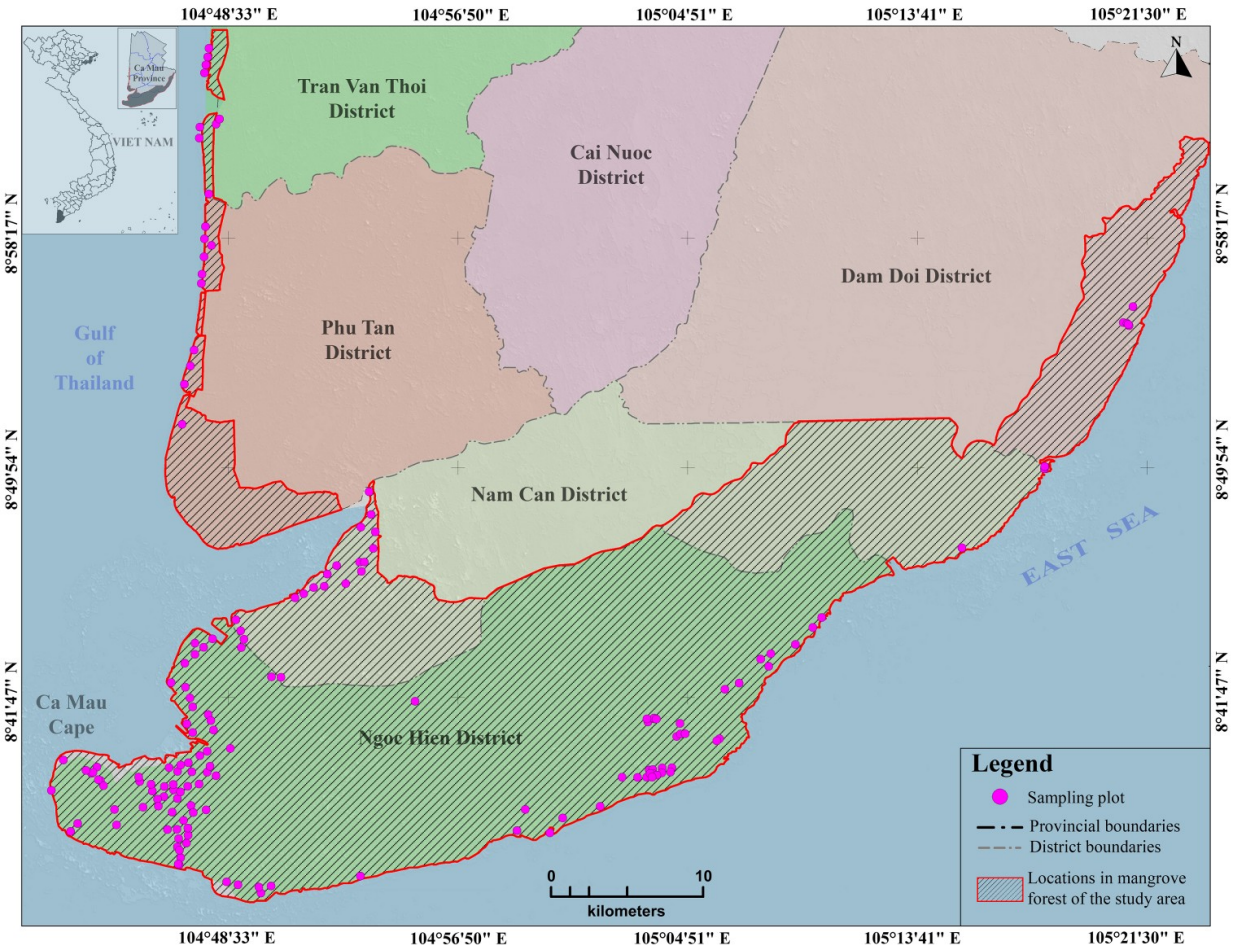
The study area in Ca Mau Province and the distribution of 158 sampling plots are presented spatially on this map. Background spatial data were collected from https://gadm.org/ and processed by the authors.

Ca Mau Province is on the southernmost coast of Vietnam, which is located on the Mekong Delta and extends between the latitudes of 8°34’N and 9°33’N and longitudes of 104°43’E and 105°25’E (**Fig 1**). Ca Mau is characterized by many rivers and canals and low and flat terrain, and it is periodically flooded. Influenced by the tropical monsoon climate near the equator, the regional weather can be divided into two seasons: the rainy (from May to November) and dry (from December to April) seasons. The study area is characterized by an average temperature of 26.5°C, an annual average rainfall of approximately 2,360 mm, an annual average evaporation of 1,022 mm and an annual average moisture of 85.6% (http://www.camau.gov.vn).

The Ca Mau mangrove forest is the second most pristine forest in Vietnam, both in terms of species composition and biomass, with an entire area of approximately 69,000 ha [2]. It is located mostly in the Ngoc Hien and Nam Can districts, and the remaining area is situated in the Dam Doi, Phu Tan, Tran Van Thoi, and U Minh districts. Most of the forest area is in the Ca Mau Cape Biosphere Reserve (41,862 ha). Surrounded by the sea and 249 km of coastline, the forest is considered an erosion barrier. The forest is also the green lung of the whole southeastern region, which plays a role in climate harmonization, ecological balancing, and environmental protection. Ca Mau has diverse mangrove species, among which the most saline-tolerant plants are in Avicenniaceae (*Avicennia alba*, *Avicennia marina*) and Rhizophoraceae (*Rhizophora apiculata*, *Rhizophora mucronata*, *Bruguiera gymnorrhiza*), *Lumnitzera racemosa*, and *Excoecaria agallocha*. Among these, Rhizophoraceae is the most popular species, so the forest is also called Rhizophora forest. Another aspect relating to the management of specific uses of certain types of mangrove forest includes the zoning of the mangrove forest into functional zones. In this regard, six distinct types of forest have been defined, namely, natural Rhizophora forest, natural mixed Avicennia/Rhizophora forest, naturally regenerated Avicennia forest, Rhizophora plantation forest (mainly *Rhizophora apiculata* and *Rhizophora mucronata*), Avicennia plantation forest (mainly *Avicennia alba* and *Avicennia marina*), and other mangroves forest and shrubs.

### Data used

#### Predictor variables from Sentinel-1A and SPOT-6 imagery

There are numerous studies on the uses of active radar at the L-band (1 GHz ÷ 2 GHz), C-band (4 GHz ÷ 8 GHz), and P-band (300 MHz ÷ 1 GHz) in estimating the AGB of mangrove forests. However, the selection of the radar bands is subject to the availability of input data from either commercial satellites or free sources. In this paper, the Sentinel-1A C-band (ground range detected product; interferometric wide swath mode; 250 km swath width; 5 × 20 m spatial resolution; VV: vertical transmit–vertical receive, and VH: vertical transmit–Horizontal receive dual polarization) was acquired on March 23, 2015. High-resolution SPOT-6 satellite imagery (Satellit Pour l’Observation de la Terre 6—February 8, 2015, with 6 m multispectral resolution capability) data were used to estimate the biomass of the mangrove forests in Ca Mau Province. The Sentinel-1A and SPOT-6 multispectral data were acquired in February/March 2015 at the same time as the field measurements were conducted. Since the spatial resolutions of Sentinel-1A (5 m x 20 m) and SPOT-6 (6 m) are technically different, the SAR data were resampled in the multispectral image to 6 m resolution by the Bilinear resampling technique, which computes new pixels using linear interpolation. This process was implemented after the preprocessing of the raw dataset by using PCI Geomatics 2018 software.

The Sentinel-1 data were preprocessed using the Sentinel-1 toolbox (S1TBX) embedded in the SNAP desktop application (version 6) from the European Space Agency (http://step.esa.int). The orbit file with accurate satellite and velocity information was also used for this step and included (i) radiometric calibration of the backscatter representation of the reflecting object (converted from digital number (DN) values to σ^o^ values); (ii) speckle filtering for speckle suppression using the Lee adaptive filter (with a window size 7×7) [13]; (iii) terrain correction using the DEM data at a 5 m spatial resolution from the Vietnam Ministry of Natural Resources and Environment to correct for the SAR geometric distortions; and projection of the images into the WGS 84 coordinate system in the UTM zone 48N projection.

The multispectral SPOT-6 data (blue, green, red, near-IR) were obtained from optical satellite sensors with a high spatial resolution of 6 meters. The study area had a cloud cover of less than 10%. The multitemporal satellite image was calibrated, and the radiation/atmospheric effects were removed by using the ATCOR (atmospheric correction) function integrated with PCI Geomatics 2018 software. The processing consisted of three parts: (i) top-of-the-atmosphere reflectance; (ii) haze removal and cloud masking; and (iii) ground reflectance atmospheric correction [29]. These images were projected into the WGS84 coordinate system/UTM zone 48N projection with ground-control points and orthorectified by using DEM data, which ensured a geometric correction accuracy of approximately ±0.5 pixels.

Even though the limitation of the C-band in interacting with the more profound components of the forest was mentioned, few works have investigated the potential uses of Sentinel-1A with a variety of optical image indices [30,31]. This study is a continuation of research on the C-band from Sentinel-1A in mangrove forest estimation, in which the combination of polarizations was proposed, such as HH, HV, HH-HV, and HH/HV, as has been suggested in many studies [1,13,15]. The structure of mangrove forests (open or closed) and the water level conditions on the acquisition day influence the volume backscatter, double-bounce backscatter and HH, HV, and VV polarizations. As a supplement, the optical imageries provide useful information about mangrove conditions by transforming spectral bands to enhance the contribution of the vegetation properties or chemical components of the leaves. A wide variety of vegetation indices that differ from each other in their transformation equations and required objectives were used in this study, as shown in (**Table 1**). Such indices have also been suggested to have significant contributions to the overall AGB estimation, as in the studies of [1,20,21,23]. Forty-two predictor variables were generated in **Table 1** and the average values (based on the centers of sample plots and plot sizes) were used as an input database for the analysis workflow, as shown in **Fig 3**.

**Table 1:** Predictor variables from Sentinel-1A and SPOT-6.

No.	Independent variables	Name/Explanations	Source
1.	**SPOT 6**	Band 1: Blue (0.455 μm– 0.525 μm)	
2.		Band 2: Green (0.530 μm– 0.590 μm)	
3.		Band 3: Red (0.625 μm– 0.695 μm)	
4.		Band 4: Near-Infrared (0.760 μm– 0.890 μm)	
5.	**Sentinel 1A**	VV (Vertical Transmit-Vertical Receive Polarizations, 3.75 to 7.5 cm wavelength)	
6.		VH (Vertical Transmit-Horizontal Receive polarizations, 3.75 to 7.5 cm wavelength)	
7.		AVERAGE_vhvv_ (polarization average)	[13]
8.		DIFF_vvvh_ (polarizations difference)	[13]
9.		MULT_vhvv_ (polarization multiply)	[13]
10.		RATIO_vvvh_ (Cross polarized ratio)	[13]
11.	**Spectral indices from SPOT 6**	ARVI (Atmospherically Resistant Vegetation Index)	[32]
12.		ATSAVI (Adjusted transformed soil-adjusted VI)	[33]
13.		AVI (Ashburn Vegetation Index)	[32]
14.		BWDRVI (Blue-wide dynamic range vegetation index)	[34]
15.		CI (Coloration Index)	[35]
16.		CIGREEN (Chlorophyll Index Green)	[36]
17.		CIRED-EDGE (Chlorophyll Red-Edge)	[37]
18.		CVI (Chlorophyll vegetation index)	[38]
19.		DVI (Difference Vegetation Index)	[39]
20.		EVI (Enhanced Vegetation Index)	[40]
21.		GI (Greenness Index)	[41]
22.		GLI (Green Leaf Index)	[42]
23.		GNDVI (Green Normalized Difference Vegetation Index)	[43]
24.		GSAVI (Green Soil Adjusted Vegetation Index)	[44]
25.		GRVI (Green Ratio Vegetation Index)	[36]
26.		I (Intensity)	[35]
27.		IF (Shape Index)	[35]
28.		MSAVI (Modified Soil Adjusted Vegetation Index)	[32]
29.		NDVI (Normalized Difference Vegetation Index)	[45]
30.		OSAVI (Optimized Soil Adjusted Vegetation Index)	[46]
31.		PBI (Plant biochemical index)	[47]
32.		PNDVI (Pan Normalized Difference Vegetation Index)	[48]
33.		PVI (Perpendicular Vegetation Index)	[49]
34.		RDVI (Renormalized Difference Vegetation Index)	[50]
35.		RI (Normalized Difference Red/Green Redness Index)	[51]
36.		SAVI (Soil Adjusted Vegetation Index)	[52]
37.		SIPI3 (Structure Intensive Pigment Index 3)	[53]
38.		TSARVI (Transformed Soil Atmospherically Resistant Vegetation Index)	[32]
39.		TSAVI (Transformed Soil Adjusted Vegetation Index)	[54]
40.		TVI (Transformed Vegetation Index)	[32]
41.		WDRVI (Wide Dynamic Range Vegetation Index)	[55]
42.		WDVI (Weighted Difference Vegetation Index)	[33]

#### Field survey dataset

The field survey was carried out from January to March 2015 in the Ca Mau district and was authorized by the Mui Ca Mau National Park and local administration. The investigations followed the guidelines issued by the Ministry of Agriculture and Rural Development with sampling plots with areas of 100 and 1,000 m^2^. The plot locations were randomly selected across the study area and provided the best description and measurement of the plot condition, canopy coverage rate, the total number of trees in each plot, average diameter, average cross-section, and tree heights. Fig 1 shows the coordinates of the center of each plot. Afterward, the AGB was statistically estimated for each plot by using single tree allometry and plot-aggregated allometry to quantify all measured trees in the plot. Two genera, Avicenniaceae (*Avicennia alba*, *Avicennia marina*) and Rhizophoraceae (*Rhizophora apiculata*, *Rhizophora mucronata*, *Bruguiera gymnorrhiza*), dominated the area, with an average density of 2,830 trees per ha, an average diameter varying from 6.9 cm to 19 cm and an estimated biomass between 40 and 340 Mg ha^-1^. The field estimation of AGB was calculated based on the estimation equations from [1,56–58], as specifically shown in Table 2.

**Table 2:** Main allometric equations for the aboveground biomass calculation of each tree species.

Forest Type	Allometric Equations
Avicenniaceae	
*Avicennia marina*	AGB = 0.308×DBH^2.11^
*Avicennia alba*	AGB = 0.131×DBH^2.46^
Rhizophoraceae	
*Rhizophora apiculata*	AGB = 0.235×DBH^2.42^
*Rhizophora mucronata*	AGB = 0.169×DBH^2.46^
*Bruguiera gymnorrhiza*	AGB = 0.186×DBH^2.31^

(DBH is the diameter at breast height).

## Background of the algorithms used

### Adaptive neuro-fuzzy inference system

**Fig 2:**
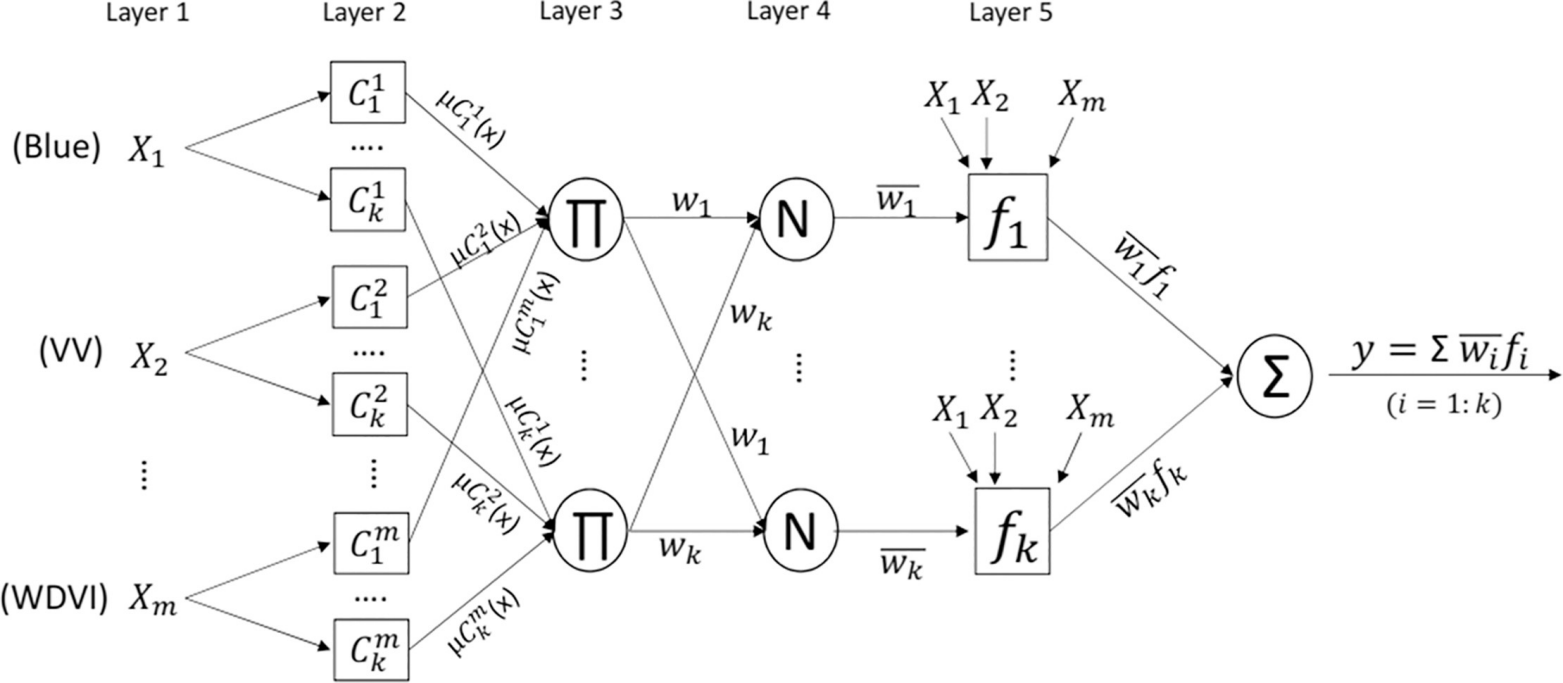
Adaptive neuro-fuzzy inference system.

Since the first study of [59], the ANFIS has been widely used to solve numerous problems, either in image classifications [27,60] or regression applications [61]. This method integrates fuzzy inference into conventional neural networks and inherits the benefits of both. A typical structure of the ANFIS is briefly described in **Fig 2**, and a more detailed explanation of each layer set can be found in various studies, such as [59].

Rule k: IF x_1_ is $C_{k}^{1}$ AND x_2_ is $C_{k}^{2}$ …. AND x_m_ is $C_{k}^{m}$ THEN P_i_ can be estimated through several steps as described in the following layers:

**Layer 1**: This layer consisted of the training data and 42 associated predictor variables (as presented in **Table 1)** or the remaining variables after the feature selection process. As a preliminary step, a clustering method was used to define the optimal clusters for this training dataset. These data were fed into **Layer 2** with the following membership function:

$$\mu C_{j}^{i}\left(x_{i}\right)=\frac{1}{1+{\left|\frac{x_{i}-c_{ij}}{a_{ij}}\right|}^{2b_{ij}}}$$ (1)

where i = 1:m, in which m is the number of input variables, and j = 1: k, where k is the number of clusters as well as the number of rules in this study. The determination of k is carried out by trial-and-error process; x_i_ indicates the input variables (42 independent variables in **Table 1** or the variables after the feature selection process); $C_{j}^{i}$ is the linguistic label, and $\mu C_{j}^{i}(x)$ is membership value that defines how much of factor (x) belongs to $C_{j}^{i}$. *P*_*i*_ is the predicted value; and *a*_*ij*_, *b*_*ij*_, and *c*_*ij*_ are adaptive parameters to be adjusted by using the ASO.

**Layer 3**: The weights in this layer are calculated by using the following equation:

$$w_{j}=\mu C_{j}^{1}\left(x_{1}\right)\times \mu C_{j}^{2}\left(x_{2}\right)\ldots \times \mu C_{j}^{m}\left(x_{m}\right)$$ (2)

**Layer 4**: Weights are normalized in this step by $\bar{w_{j}}=\frac{w_{j}}{sum(w_{j})}$

**Layer 5**: This adaptive layer takes the sum of linear functions multiplied by the normalized weights in the previous step. The equations are as follows:

$$f_{j}=\bar{w_{j}}\left(p_{0}^{j}+\sum \left(p_{j}^{k}\;x_{i}\right)\right)$$ (3)

$$Final\;summation:\;P_{i}=\sum_{j=1}^{k}f_{j}$$ (4)

$$\mathrm{RMSE}=\sqrt{\frac{1}{n}{\left(P_{i}-O_{i}\right)}^{2}}$$ (5)

where $p_{0}^{j}$ and $p_{j}^{k}$ are to be adjusted at the same time as *a*_*ij*_, *b*_*ij*_, and *c*_*ij*_ by the ASO. *O*_*i*_ is the observed value (ground-truth value). n is the training size. Eq 5 is used as the objective function for the ASO search. In general, the number of adaptive parameters is calculated based on the number of input features and the number of clusters and is presented as follows: No. of parameters = m * k * 3 + (m + 1) * k, in which 3 represents *a*_*ij*_, *b*_*ij*_, *c*_*ij*_ of the membership functions as described in Eq 1. These parameters are tuned by the ASO as described in the next section.

### Atom search optimization

First introduced by [62], the ASO is formulated based on the molecular dynamics in which all atoms in search spaces interact with each other through attraction and repulsion forces. The equilibrium stage is achieved when two forces are equal at a distance *r*_*ij*_ = 1.12*σ*. Technically, the mass of a defined atom represents a solution, where a heavier atom is better than a lighter one. Atom masses are influenced by the movement of all other atoms. The ASO can be simply described as follows:

The objective function was defined in Eq 5 to minimize the RMSE of the regression function. To solve this minimization problem, an atom population of *n* in *d* dimensional space was proposed. That was $x_{i}=[x_{i}^{1},x_{i}^{2}\ldots .x_{i}^{d}]$, *i* = 1…*n*. where *r*_*ij*_ is the Euclidean distance between atom *i* and atom *j* in *d* dimensional space. The positions of the atoms were randomly generated in the *d* dimensional space *x*_*i*_(*i* = 1..*n*), and *d* is equal to the adaptive parameters of the ANFIS. Atoms interact with each other by the force defined in Eq 6, and the total interaction is represented in Eq 7.

$$F_{\mathrm{ij}}=-\nabla U\left(r_{\mathrm{ij}}\right)=\frac{24\varepsilon }{\sigma^{2}}\left[2{\left(\frac{\sigma }{r_{\mathrm{ij}}}\right)}^{14}-{\left(\frac{\sigma }{r_{\mathrm{ij}}}\right)}^{8}\right]r_{\mathrm{ij}}$$ (6)

$$F_{i}=\sum_{j=1,j\neq i}^{N}F_{\mathrm{ij}}$$ (7)

where U(r_ij_) is the Lennard-Jones (L-J) potential between atoms i and j and σ is the length scale that denotes the collision diameter. This potential U(rβ|) controls how the atoms interact and therefore determines the positions of the atoms after each iteration.

The mass of the atoms is recalculated after each iteration by using Eq 8 and Eq 9:

$$M_{i}\left(t\right)=e^{-\frac{Fit_{i}\left(t\right)-Fit_{best}\left(t\right)}{Fit_{worst}\left(t\right)-Fit_{best}\left(t\right)}}$$ (8)

$$m_{i}\left(t\right)=\frac{M_{i}\left(t\right)}{\sum_{j=1}^{N}M_{j}\left(t\right)}$$ (9)

The interaction force F_i_ and acceleration are calculated after each iteration. From this stage, the positions and velocities of all atoms are updated by using the following:

$$v_{i}^{d}\left(t+1\right)=rand_{i}^{d}\;v_{i}^{d}\left(t\right)+a_{i}^{d}\left(t\right)$$ (10)

$$x_{i}^{d}\left(t+1\right)=x_{i}^{d}\left(t\right)+v_{i}^{d}\left(t+1\right)$$ (11)

where ${rand}_{i}^{d}$ are random values between [0] and [1].

The searching process terminates when the predefined maximum number of iterations is reached or the objective function reaches a smaller than desirable value. By integrating them into the ANFIS, the optimal parameters of the ANFIS were defined for calculating biomass for the whole study area.

### Feature selection

Feature selection has considerable impacts on the performance of classification or regression models through the elimination of irrelevant variables, handling multicollinerity [63] and might effectively boost the operation [1,27]. It is also necessary to compare classifiers in an unbiased manner. The simple Spearman correlation, which assesses monotonic relationships, i.e., linear or nonlinear, has sometimes been used, but it has some specific limitations in the nonlinear relationship. In some cases, even the Spearman coefficient indicates that two features are highly correlated in low dimensional space; i.e., they are linear or nonlinear, which can provide very different information in high-dimensional space. In this study, several feature selection methods were examined, such as Relief Attribute Evaluation [64,65], subclass evaluation [65], Correlation Attribute Evaluation [26,65], and the genetic algorithm [27,65]. Generally, the features were chosen by gradually adding more features until the performance of the classifier started to drop. The use of cross-validation to calculate precision will probably reveal that different classifiers choose different feature combinations. The features that are selected by these methods will be examined by the proposed hybrid model, and the detailed assessment is in the next section.

### Performance assessment

This study aimed to find the best fit model but to ensure that the model would not be overfitted. This was achievable by estimating the expected prediction error. Therefore, to eliminate overfitting problems, more training datasets were required, and different sampling methods were used. Typically, there are three common ways to train and validate a model: (1) The hold-out method randomly divides the points in the training set into roughly 70% for training and 30% for validation, and (2) k-fold cross-validation (CV) randomly divides the training set into k equal folds. In this case, previous studies have shown that ten-folds is the optimal number for this method [23]. (3) Leave p-out cross-validation with p equal to 1 is usually applied. Each method requires a specific size of the training set, and in this case study, the sampling size was large enough, so the hold out method was applied.

In general, for a regression study, the coefficient of determination (R^2^) is useful for explaining the explanatory power of independent predictor variables. It is a common indicator that has been used in all AGB estimation studies, such as in [1,13,15,23]. In other words, R^2^ gives a sense of how well the model can explain the input dataset. Initially, R^2^ ranges from 0 to 1, but negative values of (R^2^) are found in some cases. This situation occurs when the model is even worse than a linear regression.

On the other hand, the RMSE (Eq 5) is useful for understanding the accuracy and precision of the estimation model by comparing the predicted data (in this case, AGB) to the observation data (in situ measures in each plot). The RMSE can be used either in classification or regression as an optimal objective function of the optimization process [27]. The drawback of this value is that it is sensitive to large errors so that a preliminary screening of input data should be performed to remove any outliers. Similarly, MAE also provides an average prediction error with negatively oriented scores, which means lower values are better. Depending on the actual dataset, the MAE and RMSE might vary differently and should not be used as comparative indicators between estimation methods.

Finally, this hybrid model was benchmarked with machine learning methods that had been used in previous mangrove studies, including MLP, SVR, and RF [20]. For this current dataset, the parameters for SVR, including the kernel width (γ) and regularization (C), were defined through a grid search. On the other hand, the determination of several trees impacted the speed of search and accuracy of the result. From trial and error tests, 500 trees was the optimal parameter for this selected RF method.

### Proposed methodology for mangrove forest classification and aboveground biomass estimation

**Fig 3:**
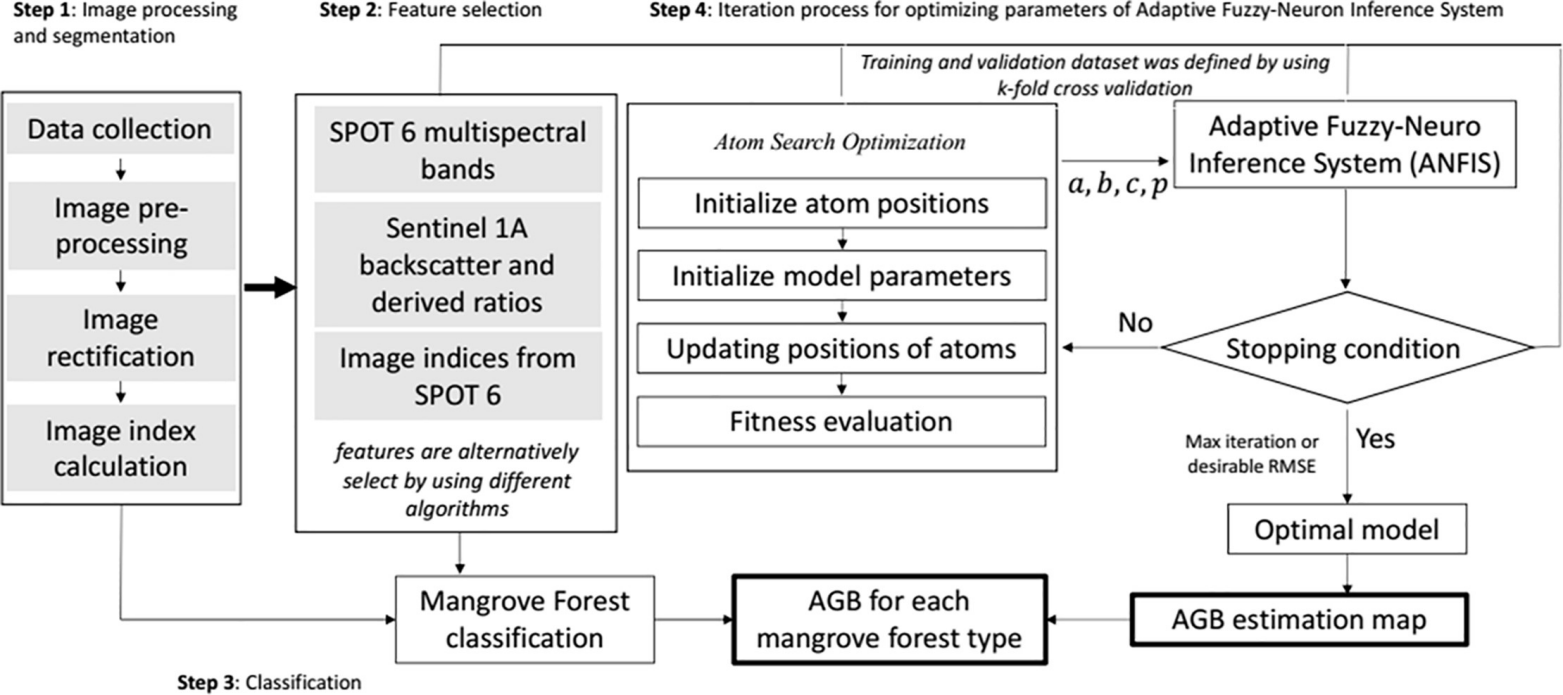
AGB estimation workflow.

This flowchart of **Fig 3** shows the step-by-step procedure starting from image processing, which was followed by the vegetation index calculation, and then the classification and AGB estimation were implemented based on the input dataset from previous steps by using the proposed machine learning method. Detailed explanations are described as follows:

**Step 1:** This step involved the preprocessing of Sentinel-1A and SPOT-6 imagery, including atmospheric correction, noise removal, image rectification, and image index calculation. This step was implemented by using SNAP, which was developed by the European Space Agency, and PCI Geomatics, a program that was created by the Canada Centre for Remote Sensing. The output from this step was a layer stack of 42 predictor variables in the UTM-WGS 84 projection with a spatial resolution of 6 m for the SPOT-6 data and 10 m for the Sentinel-1A data, which is resampled to 6 m by using SPOT-6 as the spatial reference. A short description of this step was explained in the previous section.

**Step 2:** The inclusion or exclusion of certain predictor variables is subject to the nature of datasets (forest type, growing stage, and geographical locations) and specific algorithms. This means that, among all predictor variables that can be collected for AGB, different algorithms might result in different combinations of variables based on the observed dependent ground truth values. Therefore, this step is vital for filtering out redundant features that might have negative influences on the predictive performance of the regression methods. This step is the iteration process, in which features are alternatively selected by feature selection methods. Each selected subset is used to run the proposed hybrid model and benchmarked algorithms for comparison.

**Step 3:** The input data from Steps 1 and 2 were used for the AGB estimation and the classification of the mangrove forests. Since the long-term objective of this work was to quantify the structure of each forest type in a time-series manner, the spatial distribution of each species was required. The process was carried out with the use of the ASO-ANFIS for the forest cover classification into six mangrove types, as mentioned in the previous section. The classified map was overlaid on the AGB map to extract the AGB for each type.

**Step 4**: For any selected subset of features, the ASO searched for the optimal parameters of the ANFIS, in which RMSE (Eq 1) was used as the objective function. The maximum number of iterations and the dimensional space were defined by the structure of the ANFIS, as presented in **Fig 2**. The hybrid model started with the preliminary initialization of the model parameters of the model (determination of atoms). The algorithm observes the movement of atoms, calculates the fitness value for each, and identifies the best position of atoms. The process iterates until the desirable condition is met. This process resulted in the smallest RMSE, and the fine-tuned parameters of the ANFIS were used to estimate the AGB for the entire study area in Ca Mau. For benchmarking, other methods were also examined against similar training subsets for the performance comparison. A brief description of the ASO and ANFIS is provided in the next section.

### Mangrove forest classification

The main focus of this work was to monitor the changes in AGB across the most preserved region in Vietnam, as mentioned in the previous section. For that reason, two independent processes were implemented in parallel, which were the classification of forest cover and the AGB estimation; the latter was the main focus. Therefore, the classification was a minor focus, and result accuracies could be tolerated. In this regard, PCI Geomatics was used for image segmentation with the determination of scale = 15, compactness = 0.5, and shape = 0.8 after several trials. A total of 998 polygons, which were randomly selected, were manually assigned to one of the six classes and used for model training, and 300 polygons were used for validation.

**Fig 4:**
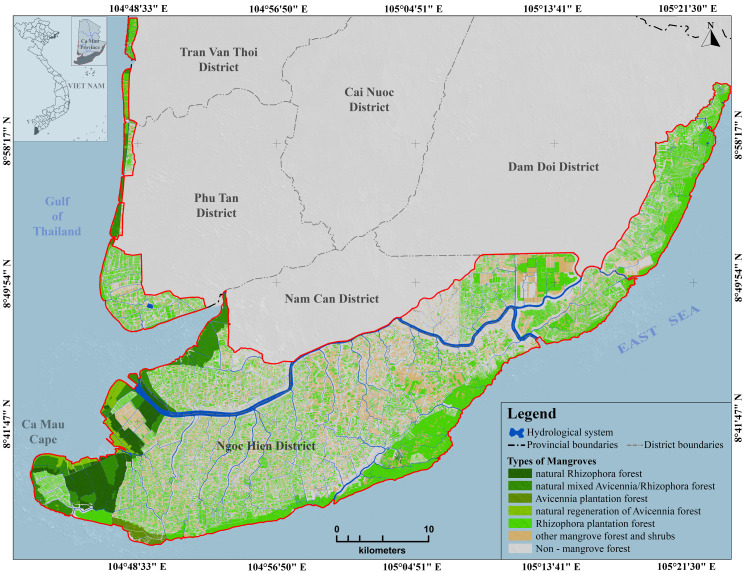
Mangrove forest classification with the use of the ASO-ANFIS. Background data were collected from https://gadm.org/ and processed by the authors.

The classification was carried out by using the ASO-ANFIS, and the result was compared to those from the most common classifiers, such as RF and SVM. The training and validation set was built from segmented objects with associated attributes from SPOT data, such as multiple spectral bands, NDVI, and texture analysis images [66]. The overall classification accuracy for the ASO-ANFIS method was 86.7%, RF was 84.8%, and SVM was 82.6% (**Table 3**). The kappa (kappa coefficients) for the ASO-ANFIS was 0.84, that for RF was 0.82, and that for the SVM was 0.81. **Fig 4** presents the results of the image classification by the ASO-ANFIS, including six mangrove classes with different densities. The natural Rhizophora forest area was 3,436.42 ha, the natural mixed Avicennia/Rhizophora forest area was 4,323.15 ha, the naturally regenerated Avicennia forest area was 1,026.21 ha, the Rhizophora plantation forest area was 26,463,05 ha, the Avicennia plantation forest area was 549.77 ha, and the other mangrove forest and shrub areas were 33,121.39 ha. The study area retains vast mangrove resources and is a prestigious World Biosphere Reserve in Vietnam.

**Table 3:** Classification accuracies.

**Training data**						
**Forest type**	Prod.Acc (%)			User Acc (%)		
	**ASO-ANFIS**	**RF**	**SVM**	**ASO-ANFIS**	**RF**	**SVM**
Natural Rhizophora forest	83.33	82.96	81.02	87.8	85.5	84.7
Natural mixed of Avicennia/Rhizophora forest	87.84	84.21	82.89	88.4	87.1	85.7
Natural regeneration of Avicennia forest	89.62	86.24	81.82	91.3	90.4	86.5
Rhizophora plantations forest	88.85	88.34	86.57	92.4	90.6	88.8
Avicennia plantations forest	88.54	85.57	80.00	91.4	89.2	86.0
other mangroves forest and shrubs	94.17	92.59	85.96	85.0	83.8	81.0
Overall Accuracy (%)						
ASO-ANFIS	RF			SVM		
89.18	87.57			85.37		
Kappa Coefficient						
ASO-ANFIS	RF			SVM		
0.87	0.85			0.83		
**Validation data**						
**Forest type**	Prod.Acc (%)			User Acc (%)		
	**ASO-ANFIS**	**RF**	**SVM**	**ASO-ANFIS**	**RF**	**SVM**
Natural Rhizophora forest	83.33	83.33	82.98	87.0	87.0	84.8
Natural mixed of Avicennia/Rhizophora forest	89.29	87.27	85.45	83.3	80.0	78.3
Natural regeneration of Avicennia forest	87.50	80.00	75.00	93.3	93.3	90.0
Rhizophora plantations forest	86.05	85.71	84.52	86.2	83.7	82.6
Avicennia plantations forest	78.95	73.68	68.42	88.2	82.4	76.5
other mangroves forest and shrubs	89.83	88.14	86.44	87.1	85.2	83.6
Overall Accuracy (%)						
ASO-ANFIS	RF			SVM		
86.76	84.70			82.58		
Kappa Coefficient						
ASO-ANFIS	RF			SVM				
0.85	0.82			0.81		

### Feature selection and AGB estimation

The training data (110 samples) and validation (48 samples) data were randomly split from 158 samples, with basic descriptive statistics such as mean = 181.9 Mg ha^-1^ and SD = 99.33 Mg ha^-1^for the training set and mean = 159.9 Mg ha^-1^ and SD = 91 Mg ha^-1^ for the validation set. Initially, the mean of the training set was higher than that of the validation set, and there was more variation in the biomass for the training set than for the validation set.

On the other hand, the configuration of the proposed model, specifically the number of weights to be tuned, is subject to the number of features that will be fed into the ANFIS. **Table 4** represents the features, which were selected by different feature selection methods (implemented by using Weka software), and the last column shows the number of parameters of the ANFIS to be tuned by the ASO-ANFIS. In this regard, the atoms (ASO algorithm) were initiated in 405-dimensional space as the result of the Relief Attribute Evaluation method, 65 as the result of CF subclass evaluation, 445 as the result of Correlation Attribute Evaluation, and 205 as the result of the GA. The search mechanism of the ASO iterated 200 times and ended up with a validated RMSE (objective function), as shown in **Table 5**. The results in **Table 5** showed that the highest R^2^ (0.58) was produced by the ASO-ANFIS, with the selected features from the genetic algorithm, which outperformed the benchmarked methods, including RF and SVR.

**Table 4:** Selected features from different methods.

Feature selection method	No of selected features	Selected features	Tunable parameters of ANFIS*
Relief Attribute Evaluation	20	ARVI, PNDVI, TSAVI, NDVI, OSAVI, SAVI, EVI, VH, ATSAVI, TVI, BWDRVI, RI, AVERAGE_vhvv_, GSAVI, GNDVI, TSARVI, CI, MULT_vhvv_, VV, I	405
CFs subclass evaluation	3	CI, GI, RATIO_vvvh_	65
Correlation Attribute Evaluation	22	CI, RI, AVERAGE_vhvv_, VV, AVI, VH, WDVI, ATSAVI, SAVI, OSAVI, NDVI, GSAVI, GNDVI, WDRVI, CVI, PNDVI, ARVI, RATIO_vvvh_, EVI, RDVI, DIFF_vvvh_, BWDRVI	445
Generic Algorithm	10	CI, EVI, IF, RATIO_vvvh_, SIPI3, TSARVI, VIN, VV, WDRVI, WDVI	205

*Tunable parameters of ANFIS are parameters of membership functions which are explained in Eq 1 and linear function in Eq 3.

**Table 5:** Statistical indicators from machine learning models by using the validation dataset.

Feature selection method	No of features	SVR			MLP			RF			RS			ASO-ANFIS		
		RMSE	MAE	*R*^2^	RMSE	MAE	*R*^2^	RMSE	MAE	*R*^2^	RMSE	MAE	*R*^2^	RMSE	MAE	*R*^2^
Relief Attribute Evaluation	20	99.31	74.50	0.33	120.23	94.76	0.40	84.41	63.77	0.48	85.55	65.49	0.45	76.37	59.93	0.51
CFs subclass evaluation	3	101.19	75.85	0.28	111.25	89.63	0.28	95.44	73.61	0.43	92.16	70.66	0.46	89.67	69.12	0.47
Correlation Attribute Evaluation	22	164.89	87.44	0.15	147.03	99.21	0.26	86.04	66.56	0.46	86.60	66.23	0.44	75.95	60.41	0.52
Generic Algorithm	10	120.05	81.70	0.24	127.21	94.37	0.34	82.23	62.53	0.50	84.41	65.72	0.48	70.88	55.46	0.58

**Fig 5:**
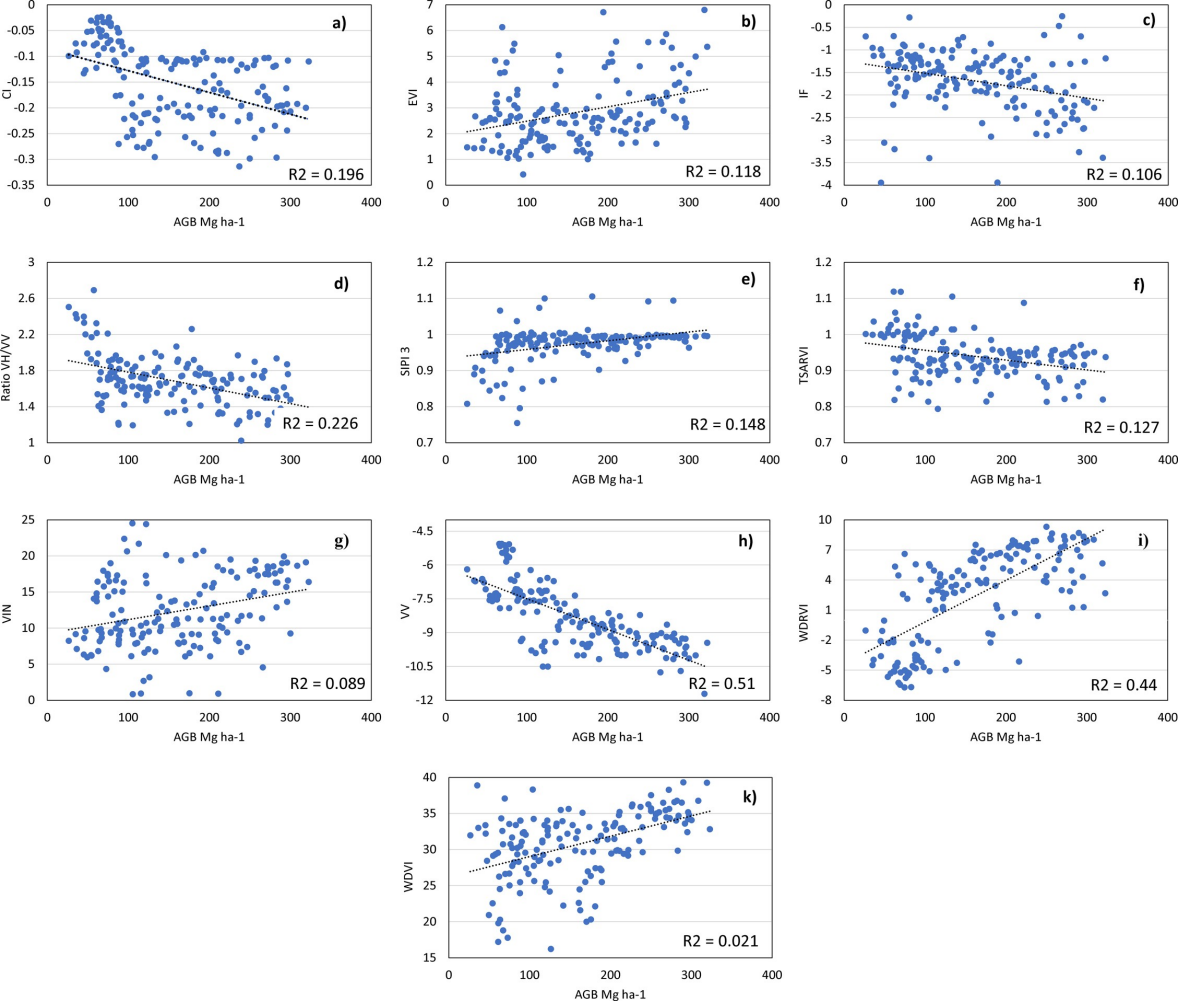
Scatter plots of 10 selected features by GA against the AGB of the sample plots: a) CI, b) EVI, c) IF, d) VH/VV ratio, e) SIPI3, f) TSARVI, g) VIN, h) VV, i) WDRVI, and k) WDVI.

The feature combinations from **Table 4** were used in the first layer of the ANFIS (**Fig 2**), and the RMSE values from the use of different repressors are shown in **Table 5**. The results showed a highest R^2^ value of 0.58, which was produced by the ASO-ANFIS with the selected features from the genetic algorithm. This process resulted in ten features, including two from the Sentinel-1A and eight spectral indices.The scatter plots are shown in **Fig 5**. The selection of VV for AGB estimation also comes along with the works of [1,67], in which VV was found to be sensitive to the increase in biomass and mangrove forest structure. The TSAVI was calculated using a soil adjustment factor [46]. WDRVI is an NDVI type, but NIR (this band was used to measure WDRVI) was rescaled by a factor ranging from 0.1–0.5. This index increased the linearity between the biomass and NIR, thus reducing the sensor saturation [68]. The contribution of VH was reflected in the ratio between VH and VV, and the remaining indices, which had been proven useful in previous studies [1,14], had a significant contribution to the overall estimation.

**Table 5** shows the estimation accuracies from the machine learning methods with different combinations of features. With ten features from GA, the ASO-ANFIS generated the highest *R*^2^ at 0.577 (rounded up to 0.58) and the smallest RMSE and MAE values of 70.88 and 55.458, respectively, among other regression methods. The two ensemble algorithms were followed by the regression methods of RF (RMSE = 82.227, *R*^2^ = 0.503) and RS (RMSE = 84.406, *R*^2^ = 0.484). SVR (*R*^2^ = 0.24) and MLP (*R*^2^ = 0.34) had the worst performance among the regression methods. However, SVR (*R*^2^ = 0.33) and MLP (*R*^2^ = 0.40) performed better with 20 selected features from the Relief Attribute Evaluation method. In addition, a test of statistical significance was also implemented, as shown in **Table 6**, in which null hypothesis H^0^ was the equality of performance. The p-values were all smaller than 0.05 (5%), so the differences were significant.

**Table 6:** Significance test between the ASO-ANFIS and the benchmarked classifiers.

	SVR	MLP	RF	RS
ASO-ANFIS	V = 68	V = 68	V = 68	V = 68
	p-value = 0.021	p-value = 0.021	p-value = 0.021	p-value = 0.021

**Fig 6:**
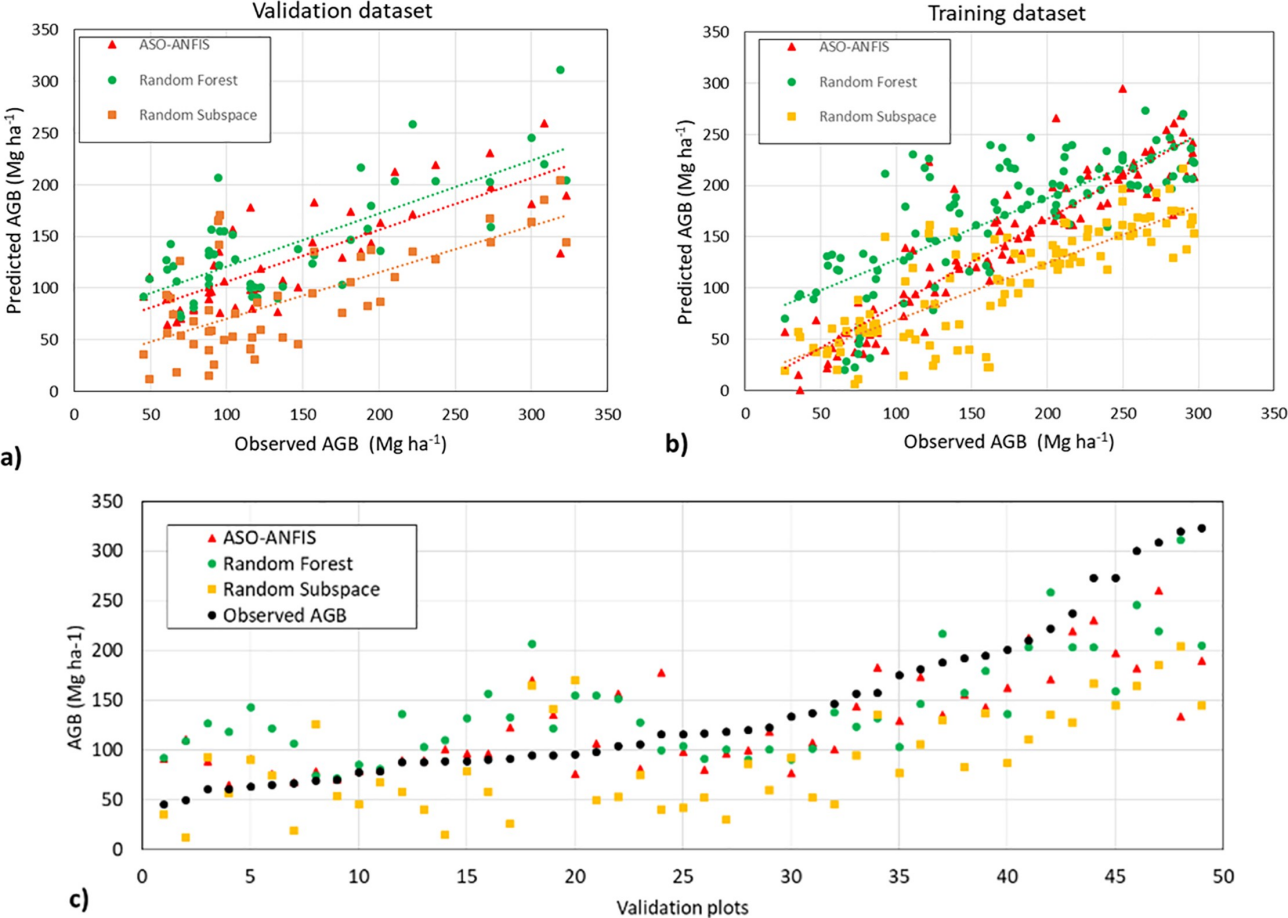
Scatter plots between the predicted AGB and observed AGB by using ten selected features from GA. a) validation dataset, b) training dataset, and c) estimated AGB in ascending order of observed AGB.

The top three highest *R*^2^ values were from the ASO-ANFI, RS, and RF by using ten selected features with the GA method, and the scatter plots between the predicted AGB and observed AGB are shown in **Fig 6**. As shown in **Fig 6**(C), the RS underestimated the AGB for the entire validation dataset. It can also be seen that for values lower than 100 Mg ha^-1^, the ASO-ANFIS and RF were likely to overestimate the AGB, and after this value, the two methods seemed to underestimate the AGB. This situation occurred due to the saturation level of the C-bands, and the spectral reflectance can only partly offset the saturation effect.

**Fig 7:**
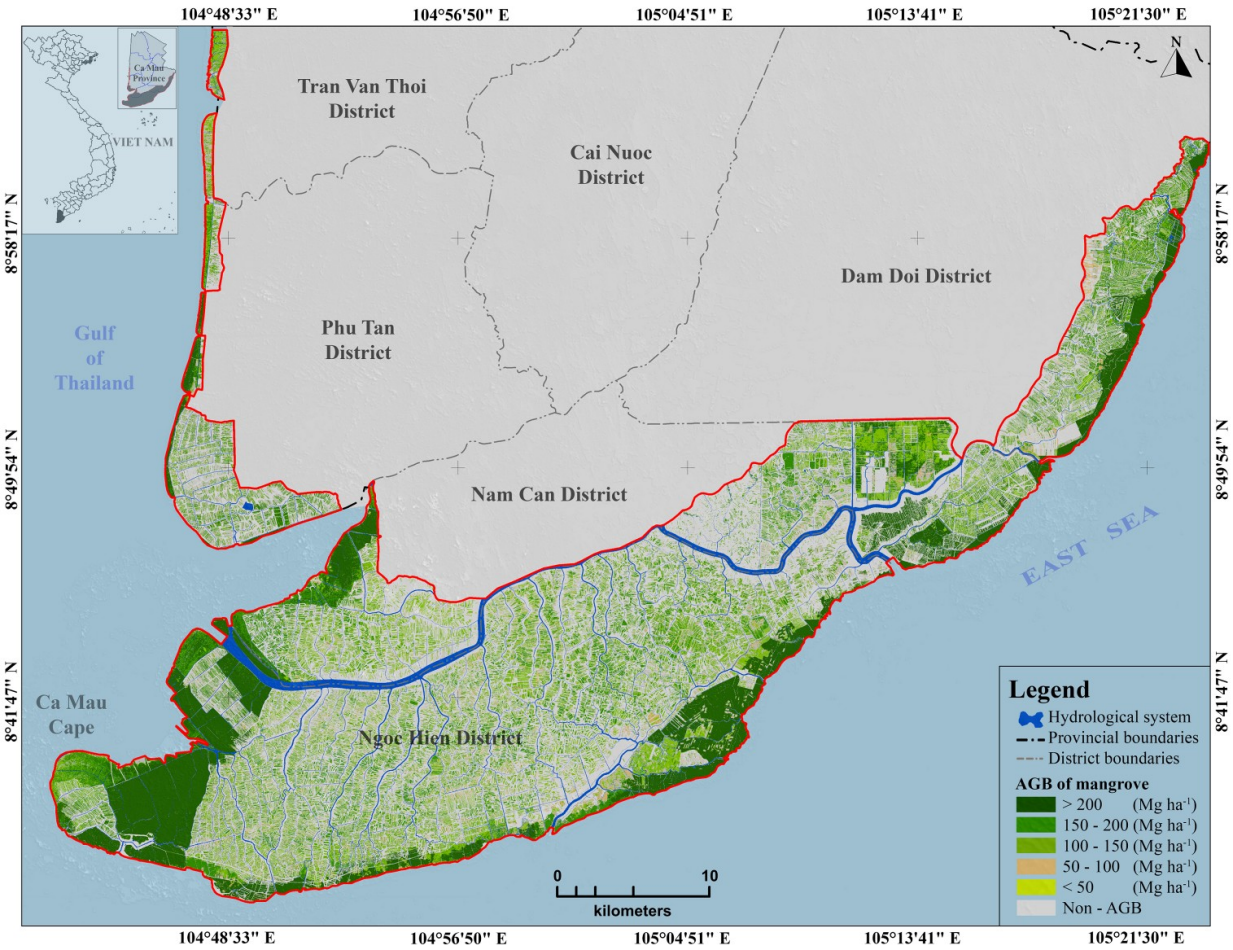
Mangrove aboveground biomass map of the study area. Background spatial data were collected from https://gadm.org/ and processed by the authors.

From the map (**Fig 7**), the AGB was grouped into the five following classes: (i) less than 50 Mg ha^-1^, (ii) from 50 to 100 Mg ha^-1^, (iii) from 100 to 150 Mg ha^-1^, (iv) from 150 to 200 Mg ha^-1^, and (v) over 200 Mg ha^-1^. With AGB values between 56.72 and 339.85 Mg ha^-1^ (average = 125.63 Mg ha^-1^), the predicted spatial pattern of the AGB value was consistent with actual observations [2,68]. The non-mangrove forested areas, such as bare land and agricultural land, were removed from the result of the AGB map.

As shown in **Fig 7** and **Table 7**, the area with an AGB over 200 Mg ha^-1^ was approximately 16,066.84 ha (approximately 23.31% of the mangrove forests of the study area), which was mainly distributed in the Rhizophora plantation forest (8,277.09 ha), natural Rhizophora forest (3,217.85 ha), natural mixed Avicennia/Rhizophora forest (2,712.74 ha), and other mangroves forest and shrubs (1,032.42 ha). Approximately 12,020.95 ha within the AGB value of 150–200 Mg ha^-1^ was found in the Rhizophora plantation forest (6,960.27 ha), other mangrove forests, shrubs (3,326.72 ha), and natural mixed Avicennia/Rhizophora forest (1,044.14 ha). The largest areas of moderate and low AGB (smaller than 150 Mg ha^-1^) were predominantly observed in the Rhizophora plantation forests and other mangrove forests and shrubs.

**Table 7:** The statistical results of the types of mangrove areas were classified into five ranges of aboveground biomass estimates.

	The area with mangrove forest density of					Total areas (ha)
	0–50	50–100	100–150	150–200	>200	
	Mg ha^-1^	Mg ha^-1^	Mg ha^-1^	Mg ha^-1^	Mg ha^-1^	
Natural Rhizophora forest	9.42	6.50	51.83	150.83	3,217.85	3,436.42
Natural mixed of Avicennia/Rhizophora forest	89.72	22.01	454.53	1,044.14	2,712.74	4,323.15
Natural regeneration of Avicennia forest	42.79	4.64	68.87	366.66	543.24	1,026.21
Rhizophora plantations forest	1,238.72	1,177.03	8,809.93	6,960.27	8,277.09	26,463.05
Avicennia plantations forest	19.58	21.65	52.71	172.32	283.50	549.77
other mangroves forest and shrubs	6,078.27	9,612.18	13,071.82	3,326.72	1,032.42	33,121.39
Total areas (ha)	7,478.51	10,844.01	22,509.69	12,020.95	16,066.84	68,920.00

The spatial distribution of the mangrove forest was observed in two regions: (i) in the western-southwestern area, this was the new alluvial land with low and flat terrain and a flooding depth of over 80 cm and (ii) the eastern coastal area of the Ngoc Hien, Nam Can and Don Doi districts. The mangrove forests here grow on acidic and highly compacted soil due to the erosion impacts from the influence of coastal currents and waves. The area was mainly Rhizophora plantation forest and mixed Avicennia/Rhizophora forest. Unlike in the western-southwestern area, in the eastern coastal area, the area of natural mangrove forests was in very low land, and there was no natural Avicennia forest (pioneering forest species encroaching on the sea).

Mapping AGB is a major concern at the global scale and for many developing countries as it is a challenging task because of the lack of field data [69]. For a given ecosystem, these maps can be used for forest monitoring, deforestation, forest degradation, and other forest-related industries, such as conservation, sustainable management, and increased carbon storage [70]. Carbon accumulation in mangrove forests is influenced by tree density, tree species, tree age, organic decomposition in soil, and regular submergence tides. Frequent tidal inundation and the degree of organic decomposition in the anaerobic environment are key factors enabling the mangrove forest in Ca Mau to become a greenhouse-gas reservoir. Therefore, the protection of the vast carbon storage in mangrove forests and on the peatlands in Ca Mau, Vietnam and throughout Asia, in general, is crucial to prevent the release of carbon dioxide and methane into the atmosphere.

### Search strategy of the ASO

**Fig 8:**
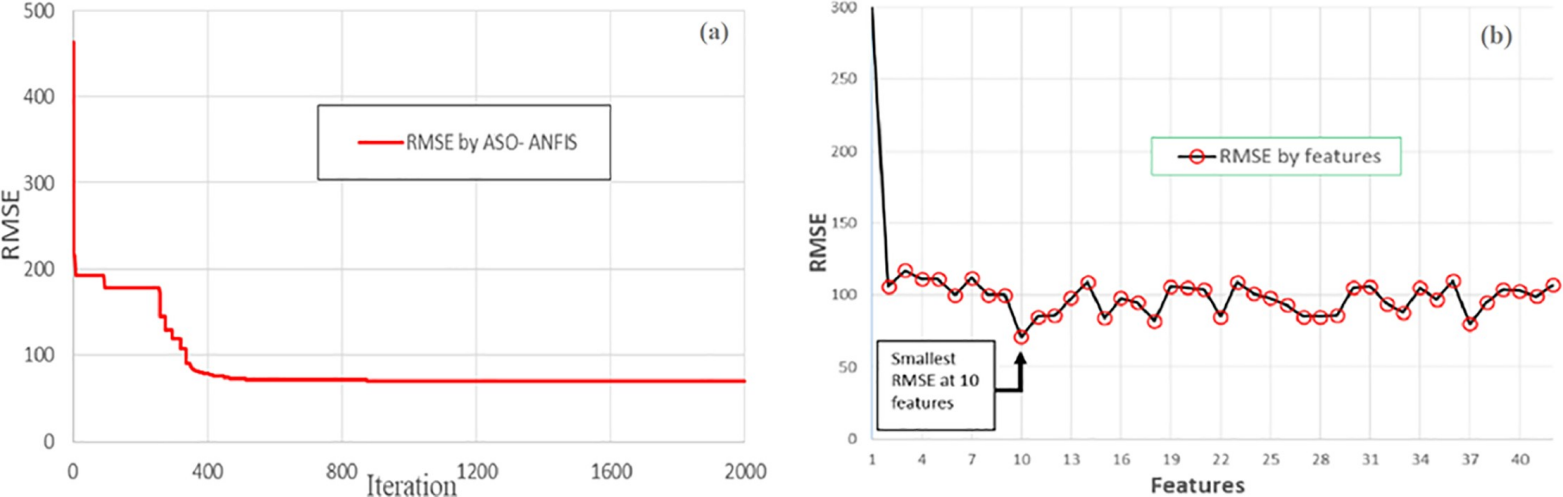
The performance of the ASO. a) Variation in the RMSE values of the validation plots with the use of the ASO and (b) variations in RMSE by the number of features using the ASO.

The hybrid model outperformed the benchmarked regression methods by comparing three common indicators, as shown in **Table 5**. This result was achieved through the robust search mechanism of the ASO in tuning parameters of the ANFIS. Indeed, the parameters of the ASO also influenced how the search operated. Fiver clusters were determined in the ANFIS and two parameters in the ASO were defined through the trial and error process. **Fig 8**(A) shows the RMSE variation curve after 2000 iterations. The exploration search can be realized by the sudden jumps in the graph (almost vertical lines in the curve) with large variation at the beginning stages.

**Fig 8**(B) shows another aspect of the ASO operation in combination with feature selection by GA. A feature selection solution was represented by a 1 x 42 dimension vector with binary values of 1 or 0, in which the selected features were represented by 1 and vice versa. For each iteration step in the GA, the ASO-ANFIS was triggered to search for the best RMSE. The horizontal axis represents the number of features that were selected during the search, regardless of how the subsets were determined. The y-axis shows the best RMSE value among the values that were generated by the ASO-ANFIS model.

### Potential uses of C bands for mangrove AGB estimation

There have been a large number of studies on the application of the L-band in AGB estimation that have investigated the relationship of backscatter signals, their transformed ratios, and the field estimation of AGB. The penetration capability of the L-band makes it useful for measuring tree cover, canopy height, and, consequently, in estimating AGB because of the correlation among these factors. The C-band has several drawbacks in comparison to the L-band in AGB applications because of its limited ability to penetrate forest canopies. The dependency of AGB estimation on the SAR wavelength has been reported in numerous studies, which have focused on the discussion of the saturation level, in which the saturation level of the C-band is typically low in AGB estimation (50–70 Mg ha^-1^) and the level of the L-band is approximately 100–150 Mg ha^-1^ [1,15]. In this study, the AGB mean of the sample plots was higher than the saturation level of the Sentinel-1A dataset, which might result in underestimation in the high AGB area. However, this situation can partly be minimized with the contribution of the optical data to the AGB estimation, as multispectral reflectance offsets the saturation effect [12,14]. The study of [71] successfully sampled plots with values higher than 300 Mg ha^-1^ by using SPOT-6 or the combination of Sentinel-1 and Sentinel-2 imagery to produce reliable AGB of approximately 200 Mg ha^-1^ [30]. The NIR, red and green bands play a key role among optical data for AGB estimation [14] because of their strong interaction with trees, and they were the main components for the measurements of the vegetation-derived features as described in **Table 4**. There have been limited studies on the combination of Sentinel-1A and SPOT-6-derived vegetation indices, and machine learning methods for AGB estimation and the uses of such datasets were the main objective of this study.

From the experiment, it could be noticed that the combination of vegetation indices from multispectral imageries with SAR improved the accuracy of AGB estimation, even though there was still underestimation (in plot samples of having AGB higher than around 120Mg ha-1) and overestimation (in plot samples of having AGB smaller than around 120Mg ha-1) of the AGB (**Fig 6**C).

This paper presented a novel model for advancing atom search optimization in searching for the parameters of the ANFIS by using 158 sample plots. The highest *R*^2^ (0.577) in this study was satisfactory compared to the (*R*^2^ = 0.28–0.44) in [72], (*R*^2^ = 0.596) in [1] with L-band PALSAR, (*R*^2^ = 0.46) in [3], even though the C-band was not as good as the L-band because of its limited ability to penetrate the canopy. Then, the proposed model (ASO-ANFIS) with ten selected features was used to generate the AGB map along the coastal area of Ca Mau Province (**Fig 7**).

## Conclusions and further remarks

Ca Mau Province is the largest reserved area for the regrowth and expansion of mangrove forests in Vietnam. The estimation results from the proposed methods showed a spatial variation in biomass that ranged from less than 40 Mg ha^-1^ to 339.85 Mg ha^-1^. This result was consistent with the in situ plot samples, with a considerable correlation between the estimated values and observed values. As the AGB is an important estimation indicator in sustainable forest management, a timely estimation of it is crucial to monitor the surface changes or potential loss and degradation of the area’s mangrove ecosystems.

The ASO significantly improved the performance of the ANFIS regression through comparison with benchmarked functions by using common statistical indicators with different combinations of features. The best values were found at RMSE = 70.882, MAE = 55.458, and R^2^ = 0.577. Another essential concept is that feature selection played an essential role in defining the most critical predictor variables before running any regression methods. This study investigated the potential uses of both optical and radar datasets, and it was found that the combination of both types of data is crucial in eliminating the saturation effect and in improving the estimation accuracy. The backscatter information from radar data and vegetation indices were evaluated to determine how they drove the changes in tree structures and associated AGB. The optimal selections are subject to the frequency of the radar dataset (X, L, P, or C bands) and spectral information of the optical data (multiple spectral bands).

This paper investigates artificial intelligence as machine learning methods and has become a trending topic because of its broad applications in almost all research fields. The increase in computational capacity and multiple sensor platforms have made geolocated data overwhelmingly available for spatial analysis. For the understanding of carbon flux, machine learning is predominantly applied to the methods used in the regression of spectral reflectance and backscatter of remotely sensed satellite imagery to the in situ measurements of AGB. This paper aimed to investigate novel hybrid machine learning algorithms for data fusion and spatial and temporal modeling for biomass estimation in the coastal area of Ca Mau Province in Vietnam. The findings are practically relevant, and the methodology is scientifically sound. This research is a novel approach and contributes to global knowledge in the field of forest cover estimation.
